# A Retrospective Analysis of Breast Cancer Mortality among Jewish and Muslim Arab Women in Israel: The Role of Sociodemographic Factors

**DOI:** 10.3390/cancers16152763

**Published:** 2024-08-05

**Authors:** Ronit Pinchas-Mizrachi, Dan Bouhnik

**Affiliations:** 1 Faculty of Nursing, Jerusalem College of Technology, Jerusalem 91160, Israel; 2 Department of Computer Science, Jerusalem College of Technology, Jerusalem 91160, Israel; bouhnik@jct.ac.il

**Keywords:** breast cancer mortality, ethnic disparities, socioeconomic status, reproductive factors, Israeli women, health inequalities

## Abstract

**Simple Summary:**

Breast cancer is a major health concern worldwide, with mortality rates varying between ethnic groups. In Israel, Jewish and Muslim Arab women have different socioeconomic backgrounds and lifestyle factors that may influence breast cancer outcomes. This study investigates disparities in breast cancer mortality between these two groups and examines how factors such as number of children, socioeconomic status, and place of residence affect mortality rates. By analyzing data from over 800,000 Israeli women over a 30-year period, the researchers aim to understand the complex interplay among ethnicity, sociodemographic factors, and breast cancer mortality. The findings of this study could help identify key risk factors and protective elements specific to each ethnic group. This information is crucial for developing targeted intervention programs to reduce disparities and improve breast cancer outcomes for both Jewish and Muslim Arab women in Israel.

**Abstract:**

Breast cancer mortality rates vary across ethnic groups in Israel, where protective factors such as high fertility and breastfeeding rates may be moderated by socioeconomic factors and mammography rates. We aim to investigate disparities in breast cancer mortality between Jewish and Muslim Arab women in Israel and examine how sociodemographic variables and number of children are associated with mortality. Our retrospective follow-up study uses data from the Israeli Central Bureau of Statistics and multivariable Cox regression models, adjusting for age, number of children, country of origin, locality size, and socioeconomic status. Compared to Jewish women, Muslim Arab women exhibited lower breast cancer mortality rates. However, after adjusting for multiple sociodemographic variables, no significant differences persisted between Jewish and Muslim Arab women. Having more than three children was associated with lower mortality among Muslim Arab women but not among Jewish women. European/American origin, larger localities, and medium socioeconomic status were associated with higher mortality. Sociodemographic factors may therefore explain the disparities in breast cancer mortality between Jewish and Muslim Arab women in Israel. Targeted intervention programs that consider the unique characteristics and risk factors of different ethnic groups are needed to reduce disparities and improve outcomes.

## 1. Introduction

Breast cancer is the second leading cause of death worldwide and the cancer with the second-highest incidence rate [1]. According to the World Health Organization, in 2020, 2.3 million females were diagnosed with breast cancer worldwide. The incidence and related mortality from breast cancer continue to grow despite remarkable advances in early detection and treatment [2,3]. In Israel, the survival rate for breast cancer is relatively high, with an estimated five-year survival rate of around 88% [4].

Breast cancer morbidity and mortality is associated with various sociodemographic risk factors. The risk of breast cancer morbidity increases with age [5]. Previous studies have found higher breast cancer mortality rates among Black women as compared to White women [6]. Among Jewish women of Ashkenazi descent, genetic characteristics have been found that are associated with breast cancer morbidity [7]. In a previous study we conducted, lower breast cancer mortality rates were found among women living in non-urban areas as compared to women living in urban areas [8].

Various other factors affect breast cancer incidence. Women of lower socioeconomic status tend to develop breast cancer less frequently than women of higher socioeconomic status [9,10], but they face a higher risk of breast cancer mortality [2] and worse survival outcomes [11,12]. They also tend to undergo mammography screening less frequently [13,14] and are at a higher risk of late-stage diagnosis [15,16]. Reproductive factors such as young maternal age at first birth [17,18], hormonal treatments [19,20], pregnancy and childbirth [21,22], and breastfeeding [21,23,24] influence breast cancer incidence. Breastfeeding and a long duration of breastfeeding have been found to be protective factors against breast cancer morbidity [23,24]. Breastfeeding can help reduce the short-term increase in breast cancer risk following pregnancy and provides additional protective benefits over time [21]. Women of lower socioeconomic status tend to breastfeed less [25].

As of 2022, Arab citizens of Israel constitute approximately 21.1% of the total population [26]. They tend to live in small- and medium-sized localities rather than large ones [27]. Compared to Jewish women, they are, on average, younger at first birth [28], less likely to use hormonal medications [29], undergo mammography [30], or enter the labor market [31] but are more likely to breastfeed for longer [32,33]. Historically, Israel’s Arab Muslim population has been characterized by particularly high fertility rates, but these have decreased over the years [34]. Previous studies have found that Arab women have lower breast cancer incidence and mortality than do Jewish women, though these gaps have recently been narrowing [29,35]. In summary, the Arab community is characterized by factors that can offer protection against breast cancer but also by factors that may negatively impact survival rates. Additionally, all-cause mortality rates are higher among Arabs as compared to Jewish women.

In this study, we aim to investigate breast cancer mortality disparities between Jewish and Muslim Arab women in Israel. Our study examines how sociodemographic factors (age, socioeconomic status, locality size, education) and number of children are associated with breast cancer mortality in each ethnic group. Elucidating the interplay of ethnicity, sociodemographic factors, reproductive history, and breast cancer mortality in Israel offers insight toward reducing disparities and improving outcomes.

## 2. Materials and Methods

We conducted a retrospective follow-up study on breast cancer mortality among Israeli women over a 30-year period, from 1 January 1990, to 31 December 2020. The data for this study were collected by the Israeli Central Bureau of Statistics from the Population Registry, Education Registry, and Ministry of Health. The study group included 817,445 Israeli women born between 1940 and 1960, of whom 743,090 were Jewish (90.9%) and 74,355 were Muslim Arab (9.1%).

Data on birth year, ethnicity (Jewish/Arab), religion, country of origin, number of children, and locality size were collected from the Population Registry. The number of children was dichotomized into “3 children or less” and “more than 3 children”. The “country of origin” variable was defined by the woman’s father’s birth country in one of three categories: 1. Israel, 2. Asia/Africa, or 3. Europe/America/Australia. Locality size was dichotomized into small (up to 20,000 inhabitants) and large (more than 20,000 inhabitants).

The socioeconomic status variable was constructed by combining two variables: education (obtained from the Central Bureau of Statistics’ Education Registry, which is based on educational data from various sources such as educational institutions, administrative files, surveys, censuses, administrative data, and more) and the socioeconomic status of residential areas (for the 20% of the population whose education data were missing). The categorization was based on the distribution of these variables in Israeli society. High socioeconomic status was determined by 15 or more years of education or a residential area socioeconomic score of 8–10. Medium status was determined by 11–14 years of education or a residential area score of 6–7. Low status was determined by up to 10 years of education or a residential area score of 1–5.

The outcome variable was breast cancer mortality, and death year was obtained from the Ministry of Health. For all variables except education, missing data were below one percent.

First, we examined the distributions for age, number of children, country of origin, locality size, and socioeconomic status, and analyzed differences between the groups relative to these variables. The statistical significance of differences was calculated using the Chi-square test for categorical variables and the *t*-test for age (Table 1).

Next, we examined mortality rates per 10,000 women by ethnicity, number of children, country of origin, locality size, and socioeconomic status. In assessing the disparities’ effect size between groups and their statistical significance, the Adjusted Hazard Ratio (AHR) for breast cancer mortality was calculated after adjusting for age (Table 2).

Then, via regression and adjusted Kaplan–Meier curves, we created multivariable models to evaluate the relationship between ethnicity and breast cancer mortality. The first model included age, ethnicity, number of children, and country of origin. The second model included those four variables and locality size. The third model included all the variables of the second model, as well as socioeconomic status (Table 3).

We found significant interactions (*p* < 0.001) among ethnicity, socioeconomic status, and number of children and breast cancer mortality. We found significant interactions between ethnicity and socioeconomic status, as well as between ethnicity and number of children and the prediction of breast cancer mortality. Therefore, we decided to separately examine the role of each of the following in predicting breast cancer mortality among Jewish and Muslim Arab women: number of children, country of origin, locality size, and socioeconomic status (Table 4).

It is important to note that for individuals who immigrated from Israel during the follow-up period and did not return by the end of the follow-up, and for whom information on their death was not obtained, survival data were calculated up to the year in which they immigrated from Israel. In other words, such individuals contributed survival years until the year they left Israel.

## 3. Results

Significant differences were found between Jewish and Muslim Arab women concerning age, number of children, country of origin, locality size, and socioeconomic status (*p* < 0.001).

**Figure 1:**
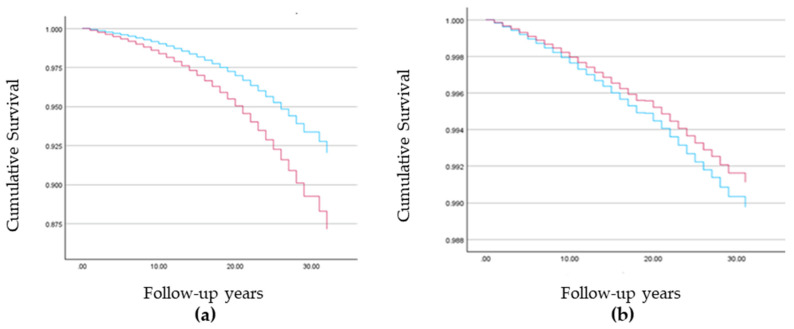
Kaplan–Meier survivor curves among the study population: Jewish women (blue line) and Muslim Arab women (red line): (**a**) All-cause mortality rates and (**b**) breast cancer mortality rates.

Higher all-cause mortality rates were found among Muslim Arab women as compared to Jewish women (AHR = 1.658, 99% CI (1.612, 1.704)) but lower breast cancer mortality rates were found among Muslim Arab women (AHR = 0.866, 99% CI (0.778, 0.964)) (Figure 1). After adjusting for age, lower breast cancer mortality rates were found among women with more than three children (AHR = 0.902; 99% CI (0.845–0.962)), those of Asian/African origin as compared to European/American origin (AHR = 1.109, 99% CI (1.031–1.192)) and Israeli origin (AHR = 1.068, 99% CI (0.997–1.143), *p* = 0.013), those living in smaller localities (AHR = 1.229, 99% CI (1.136, 1.330)), and those with low socioeconomic status as compared to medium socioeconomic status (AHR = 1.179, 99% CI = 1.101, 1.252). No significant difference was found between high and low socioeconomic status (AHR = 1.019, 99% CI = 0.945, 1.098) (Table 2, Figure 1).

As displayed in Table 3, Model 1 accounted for age, ethnicity, number of children, and country of origin. It found lower breast cancer mortality rates among Muslim Arab women (HR = 0.815; 99% CI (0.722, 0.920)), those with more than three children (AHR = 0.925; 99% CI (0.963, 0.992)), and those of Asian/African origin as compared to European/American origin (AHR = 1.166, 99% CI (1.078–1.260)). No significant differences were found between Israeli and Asian/African origin. Model 2 accounted for the same variables, along with locality size. Likewise, it found lower breast cancer mortality rates among Muslim Arab women (HR = 0.849; 99% CI (0.752, 0.960)), those with more than three children (AHR = 0.938; 99% CI (0.875, 1.007), *p* = 0.019), those of Asian/African origin as compared to European/American origin (AHR = 1.181, 99% CI (1.092, 1.277)), and those living in smaller localities (AHR = 1.219, 99% CI (1.124, 1.322)). Similarly, no significant differences were found between Israeli and Asian/African origin. Model 3 accounted for the same variables as Model 2, along with socioeconomic status. It found no significant difference between Muslim Arab and Jewish women. Lower breast cancer mortality rates were found among those with more than three children (AHR = 0.924; 99% CI (0.861, 0.992)), those of Asian/African origin as compared to European/American origin (AHR = 1.173, 99% CI (1.083, 1.270)) and Israeli origin (AHR = 1.060, 99% CI (0.985, 1.140), *p* = 0.004), those living in smaller localities (AHR = 1.189, 99% CI (1.095, 1.291)), and those with low socioeconomic status as compared to medium socioeconomic status (AHR = 1.140, 99% CI (1.060, 1.227)). No significant difference was found between high and low socioeconomic status (AHR = 0.970, 99% CI (0.893, 1.364)).

We found significant interactions between ethnicity and both socioeconomic status and number of children and the prediction of breast cancer mortality. Accordingly, the study population was divided into two groups, Muslim Arab women and Jewish women, by Cox regression and adjusted Kaplan–Meier. The models were built for each group with the following variables being entered separately with age: number of children, country of origin, locality size and socioeconomic status.

**Figure 2:**
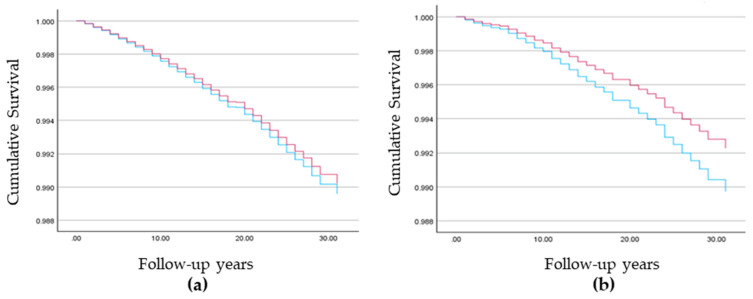
Kaplan–Meier survivor curves among the study population: 0–3 children (blue line) and >3 children (red line): (**a**) Breast cancer mortality among Jewish women and (**b**) Muslim Arab women.

Among Muslim Arab women, lower breast cancer mortality rates were found among those with more than three children (AHR = 0.751; 99% CI (0.609, 0.927)) and higher rates among those with high (AHR = 1.591, 99% CI (1.052, 2.631)) and medium socioeconomic status (AHR = 1.309, 99% CI (1.015, 1.796)) as compared to low. Country of origin and locality size were not significant predictors (Table 4, Figure 2).

**Figure 3:**
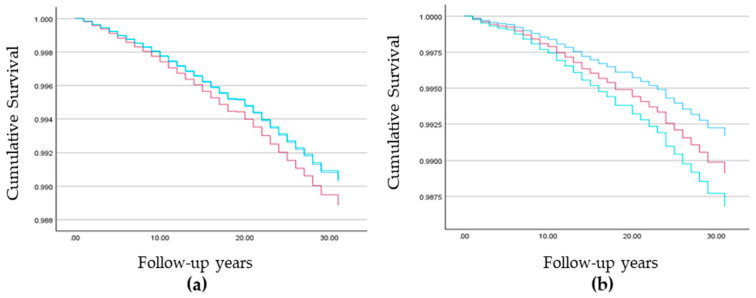
Kaplan–Meier survivor curves among the study population: low (blue line), medium (red line), and high socioeconomic status (green line): (**a**) Breast cancer mortality among Jewish women and (**b**) Muslim Arab women.

Among Jewish women, number of children was not a significant predictor. Higher breast cancer mortality rates were found among those of European/American (AHR = 1.185, 99% CI (1.096, 1.282)) and Israeli origin (AHR = 1.080, 99% CI (1.001, 1.146)) as compared to those of Asian/African origin, those living in larger localities (AHR = 1.217, 99% CI (1.115, 1.329)), and those with medium socioeconomic status (AHR = 1.146, 99% CI (1.064, 1.235)) as compared to low socioeconomic status. No significant difference was found between high and low socioeconomic status (AHR = 0.988, 99% CI (0.912, 1.071)) (Table 4, Figure 2 and Figure 3).

Overall, Muslim Arab women had lower breast cancer mortality rates as compared to Jewish women. Having more than three children was associated with lower rates in the overall population and among Muslim Arab women but not among Jewish women. Women of European/American origin had higher rates as compared to those of Asian/African origin, consistently among Jewish women but not among Muslim Arab women. Living in larger localities was associated with higher rates, consistently among Jewish women but not among Muslim Arab women. Medium socioeconomic status was associated with higher rates as compared to low status. Among Muslim Arab women, both high and medium status were associated with higher rates, while among Jewish women, only medium status was associated with higher rates as compared to low rate.

## 4. Discussion

This study investigated differences in breast cancer mortality between two groups and examined how factors such as number of children, socioeconomic status, and place of residence affect mortality rates. By analyzing data from over 800,000 Israeli women over a 30-year period, the researchers’ goal was to understand the complex interrelationships among ethnicity, sociodemographic factors and breast cancer mortality.

Similar to previous studies among Muslim Arab women, we also found higher fertility rates [34], lower socioeconomic status [26], and higher tendency to live in smaller localities [27] as compared to Jewish women. Previous studies have also found genetic factors associated with breast cancer morbidity and mortality, with specific genetic characteristics identified among Jewish women of Ashkenazi descent [6,7]. In this study, lower breast cancer mortality was found among Jewish women whose countries of origin were Asia/Africa as compared to those originating from America/Europe. However, this finding was not observed among Muslim Arab women, possibly due to this community’s homogeneity regarding country of origin.

Furthermore, this study found higher all-cause mortality rates among Arab women as compared to Jewish women, while breast cancer mortality was higher in Jewish women [29,35]. However, these disparities were not significant after adjusting for variables such as number of children, country of origin, locality size, and socioeconomic status. This may indicate that the differences in mortality between groups can be explained by those variables.

Previous studies have found that women of lower socioeconomic status (SES) have higher breast cancer mortality rates as compared to women of higher SES [2,11]. This disparity has been attributed to lower rates of mammography screening [13,14] later-stage diagnosis [15,16], and worse survival outcomes among women of lower SES [11]. However, a meta-analysis by Taheri et al. [12] suggests that the association between SES and breast cancer mortality rates may not be as strong as previously thought. In contrast to these findings, our study found higher breast cancer mortality rates among women of medium SES as compared to low SES, both among Jewish and Muslim Arab women. This can be explained by Israel’s National Health Insurance Law, which allows all women to undergo mammography, significantly reducing the barrier to screening. Furthermore, the law provides women of low SES with access to treatment, potentially leading to higher survival rates.

Coinciding with our previous study [8], we found lower breast cancer mortality rates among Jewish women residing in non-urban areas as compared to those living in urban areas. This finding was not observed among Muslim Arab women, possibly due to differences in quality of life and air pollution exposure between Jewish and Arab communities [35].

Furthuremore, our study found intriguing results regarding breast cancer mortality rates among women of high socioeconomic status as compared to women of low socioeconomic status. In Jewish women, no significant differences in breast cancer mortality were found between high and low socioeconomic status groups. Conversely, in Arab Muslim women, a gradient of increasing breast cancer mortality risk was observed with higher socioeconomic status as compared to women of low socioeconomic status. This finding could be explained by the fact that Jewish women of high socioeconomic status have greater access to resources for extending survival from the disease, while Arab women of similar socioeconomic status have less access. It can be hypothesized that Arab women of high socioeconomic status are more likely to enter the labor market, which is still less common in Arab society [31]. This entry into the workforce may be associated with relatively lower breastfeeding rates. [33]. Furthermore, it can be speculated that Arab women of high socioeconomic status are less likely to breastfeed and less likely to marry and have their first child at a young age, which may increase the risk of morbidity. Previous studies have shown that breastfeeding is associated with a reduced risk of breast cancer [23], and thus, lower breastfeeding rates among Arab women of high socioeconomic status may partially explain the higher breast cancer mortality rates in this group.

Full-term pregnancy and childbirth have been found in previous studies to be protective factors against breast cancer morbidity in the long term [21,22]. However, regarding predictors of breast cancer mortality, we found a high number of children was protective in the general population and among Muslim Arab women but not among Jewish women. This phenomenon can be explained by several factors. Compared to Arab women in Israel, Jewish women tend to have lower breastfeeding rates, older age at breastfeeding, and older maternal age at first birth [28,33]. Arab women are more likely to breastfeed and report fewer difficulties and barriers to breastfeeding [32,33]. Arab women are less likely to enter the labor market [31] and less likely to use hormonal medications [29]. These factors, along with the tendency of Arab women to have a lower maternal age at first birth and to breastfeed more, may contribute to the lack of protective effect of a high number of children against breast cancer mortality in Jewish women as compared to Arab women in Israel.

This study has several limitations. First, it is based on administrative data. Cause of death was determined based on information collected from death certificates by the Ministry of Health. Another limitation was the lack of data on breast cancer incidence and prevalence rates among the studied groups. Such data would have provided a more comprehensive picture and enabled the evaluation of mortality disparities in relation to morbidity and survival disparities.

An additional limitation of our study is the use of parity as a dichotomous variable, with a single threshold of three or more children. While this threshold aligns with the average Israeli fertility rates, exploring different parity levels could provide more nuanced insights. Future research should consider a more granular analysis of parity to better understand how varying fertility rates in different populations might influence breast cancer outcomes.

The absence of education data for a relatively large portion of the population led the authors to use a composite variable of socioeconomic status instead.

## 5. Conclusions

This study’s findings underscore the complex interplay of ethnicity, socioeconomic status, and reproductive factors in breast cancer mortality among Israeli women. The differential impact of risk factors between Jewish and Muslim Arab populations emphasizes the need for culturally sensitive, group-specific interventions. The paradoxical relationship between socioeconomic status and breast cancer mortality in the Israeli context, particularly among Muslim Arab women, warrants further investigation. Moreover, the varying protective effect of parity between ethnic groups suggests intricate interactions with factors such as breastfeeding practices and age at first birth. These results highlight the importance of developing nuanced research approaches and tailored prevention strategies that account for the specific risk profiles of different ethnic and socioeconomic groups. Future studies should aim to elucidate these complex relationships to inform more effective, targeted public health interventions and policies, ultimately reducing breast cancer mortality disparities in Israel’s diverse population.

## Figures and Tables

**Table 1:** Distribution of research variables among Jewish and Muslim Arab women.

		Jewish (*n* = 743,090)	Muslim Arab (*n* = 74,355)	*p*
Age		39.08 (5.66)	37.99 (5.90)	<0.001
Number of children		2.45 (1.95)	4.87 (3.54)	<0.001
Country of origin	Asia/Africa	33.4%	4.0%	<0.001
	Europe/America	44.0%	1.4%	
	Israel	22.6%	94.6%	
Locality size	Small	84.6%	55.3%	<0.001
	Large	15.4%	44.7%	
Socioeconomic status	Low	23.7%	84.7%	<0.001
	Medium	43.2%	11.8%	
	High	33.1%	3.6%	

**Table 2:** Breast cancer mortality rate by research variables among study population (*n* = 817,455).

		Breast Cancer Mortality per 10,000 Women	AHR (Age-Adjusted Hazard Ratio), 99% CI	*p*
Ethnicity	Jewish	105.40	1.00	
	Muslim Arab	84.33	0.866 (0.778–0.964)	<0.001
Number of children	0–3	106.54	1.00	
	>3	95.19	0.902 (0.845–0.962)	<0.001
Country of origin	Asia/Africa	96.47	1.00	
	Israel	105.06	1.068 (0.997–1.143)	0.013
	Europe/America	107.59	1.109 (1.031–1.192)	<0.001
Locality size	Small	84.30	1.00	
	Large	107.70	1.229 (1.136–1.330)	<0.001
Socioeconomic status	Low	99.96	1.00	
	Medium	111.94	1.179 (1.101–1.262)	<0.001
	High	97.27	1.019 (0.945–1.098)	0.526

**Table 3:** Multivariate Cox models for breast cancer mortality prediction among study population (*n* = 817,445).

		Model 1	Model 2	Model 3
		HR (99% CI)	HR (99% CI)	HR (99% CI)
Ethnicity	Jewish	1.00	1.00	1.00
	Muslim Arab	0.815 (0.722–0.920)	0.849 (0.752–0.960)	0.904 (0.793–1.031)
Number of children	0–3	1.00	1.00	1.00
	>3	0.925 (0.963–0.992)	0.938 (0.875–1.007)	0.924 (0.861–0.992)
Country of origin	Asia/Africa	1.00	1.00	1.00
	Israel	1.044 (0.972–1.120)	1.046 (0.974–1.123)	1.060 (0.985–1.140)
	Europe/America	1.166 (1.078–1.260)	1.181 (1.092–1.277)	1.173 (1.083–1.270)
Locality size	Small		1.00	1.00
	Large		1.219 (1.124–1.322)	1.189 (1.095–1.291)
Socioeconomic status	Low			1.00
	Medium			1.140 (1.060–1.227)
	High			0.970 (0.893–1.364)

**Table 4:** Breast cancer mortality rate by research variables among Jewish (*n* = 743,090) and Muslim Arab women (*n* = 74,355).

		Model A: Jewish (*n* = 743,090)	Model B: Muslim Arab (*n* = 74,355)
		Age adjusted HR (99% CI)	Age adjusted HR (99% CI)
Number of children	0–3	1.00	1.00
	>3	0.960 (0.896–1.028)	0.751 (0.609–0.927)
Country of origin	Asia/Africa	1.00	1.00
	Israel	1.08 (1.001–1.146)	0.651 (0.159–4.466)
	Europe/America	1.185 (1.096–1.282)	0.703 (0.373–1.326)
Locality size	Small	1.00	1.00
	Large	1.217 (1.115–1.329)	1.177 (0.954–1.451)
Socioeconomic status	Low	1.00	1.00
	Medium	1.146 (1.064–1.235)	1.309 (1.015–1.796)
	High	0.988 (0.912–1.071)	1.591 (1.052–2.613)

## Data Availability

Data are available upon reasonable request.
